# Conservative Approach in the Management of Radicular Cyst in a Child: Case Report

**DOI:** 10.1155/2013/123148

**Published:** 2013-02-17

**Authors:** Narendra Varma Penumatsa, Srinivas Nallanchakrava, Radhika Muppa, Arthi Dandempally, Priyanaka Panthula

**Affiliations:** Department of Pediatric Dentistry, Panineeya Mahavidyalaya Institute of Dental Sciences & Research Centre, Dilsukhnagar, Hyderabad, Andhra Pradesh 500060, India

## Abstract

Radicular cyst is the most common odontogenic cystic lesion of inflammatory origin. It is also known as periapical cyst, apical periodontal cyst, root end cyst, or dental cyst. It arises from epithelial residues in the periodontal ligament as a result of inflammation. The inflammation usually follows the death of dental pulp. This paper presents a case report of a patient with radicular cyst associated with a primary molar.

## 1. Introduction

Radicular cyst is an inflammatory jaw cyst originating from epithelial remnants of the periodontal ligament as a result of inflammation that is generally a consequence of pulp necrosis. The resulting cyst commonly involves the apex of the affected tooth [1]. They are the most common of all jaw cysts and comprise about 52.3% [2] to 68% [3] of all cysts affecting the mandible [4]. Caries is the most frequent aetiological factor of radicular cyst in the primary dentition [5]. They also result from the traumatic injuries to the primary teeth [6].

Very few cases are seen in the first decade, after which there is a fairly steep rise, with a peak frequency in the third decade. Radicular cysts are rare in the primary dentition, representing only 0.5–3.3% of the total number in both primary and permanent dentitions [7]. The male preponderance occurs essentially in the fourth and fifth decades. The lower frequency in females, which has also been reported by other workers, may be because they are less likely to neglect their teeth [8]. These cysts are slow growing and asymptomatic unless secondarily infected. Extraction or endodontic treatment of the affected tooth is required when clinical and radiographic characteristics indicate a periapical inflammatory lesion. The normal treatments for radicular cysts include total enucleation in the case of small lesions, marsupialisation for decompression of larger cysts, or a combination of the two techniques. Inflammatory cysts do not recur after adequate treatment.

## 2. Case Report

An 8-year-old male patient reported to the department of pedodontics and preventive dentistry, PMVIDS, Hyderabad, with a chief complaint of extra oral swelling on the left lower back region of the jaw since 2 months. Past dental history revealed that he had undergone extraction in the same region 7 days ago.

**Figure 1:**
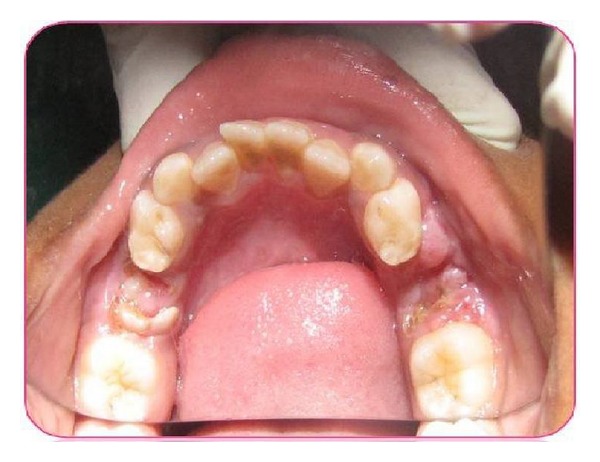
Preoperative picture showing grossly decayed mandibular left primary second molar.

**Figure 2:**
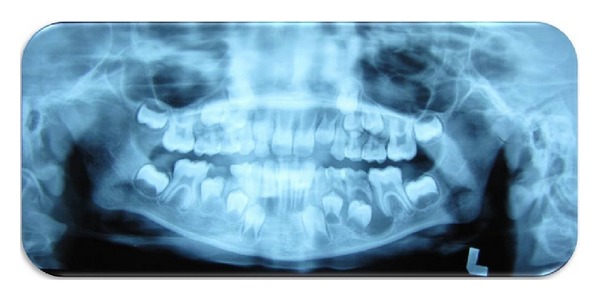
Orthopantomograph showing mandibular left primary second molar associated with periapical radiolucency.

On extraoral examination, a well-defined, nontender, hard bony swelling was noticed on the left side of the body of the mandible measuring 2 × 3 cm in size. Intraoral examination revealed grossly decayed left mandibular primary second molar with buccal cortical plate expansion (Figure 1). Orthopantomograph revealed a single well-defined periapical radiolucency measuring about 1.5 × 2 cm in size involving tooth bud of 35 which was in Nolla's stage 4 (two thirds crown completed) (Figure 2). Based on history, clinical and radiographic examination, a provisional diagnosis of radicular cyst associated with second mandibular left primary molar was made.

## 3. Treatment

**Figure 3:**
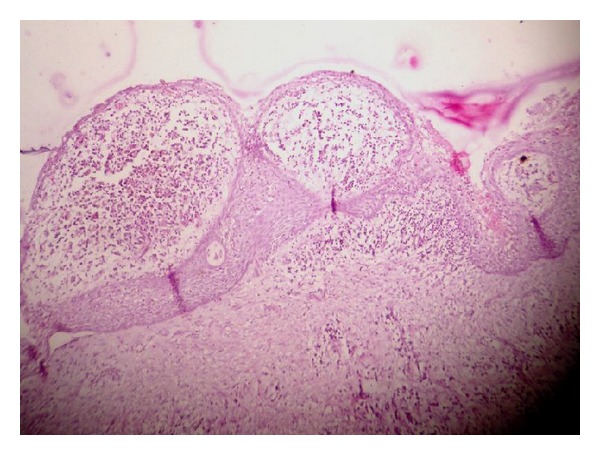
Histological view of radicular cyst.

Conservative treatment was planned to save premolar tooth bud; treatment plan included extraction of left mandibular first and second molars and followed by marsupialisation under local anaesthesia. A tissue specimen was then sent for the histological examination which confirmed our provisional diagnosis of a radicular cyst. Findings of the histological view showed stratified squamous epithelium with underlying connective tissue. Epithelium showed an arcading pattern (Figure 3). Regular followup of the case was done. Postextraction healing was uneventful.

## 4. Followup

**Figure 4:**
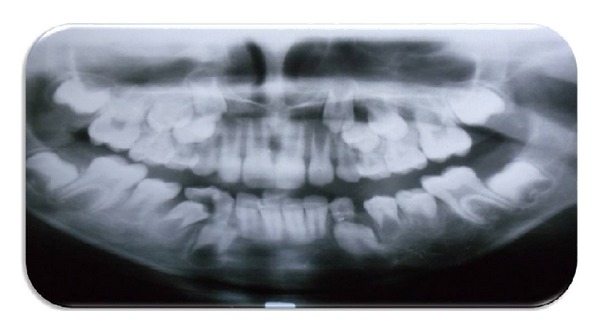
Orthopantomograph showing erupted mandibular left first and second premolars.

**Figure 5:**
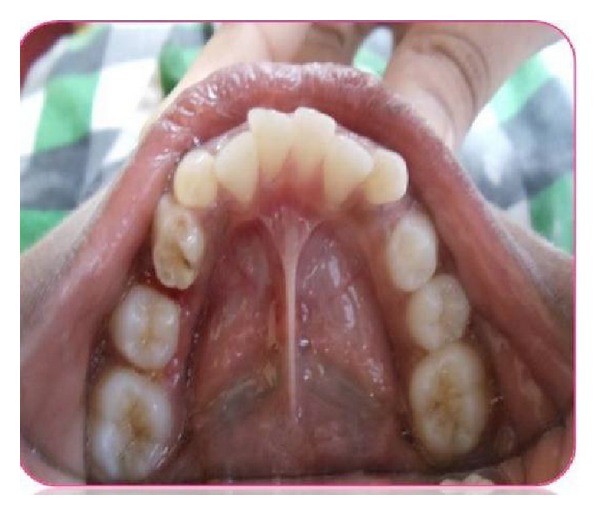
Postoperative picture after 8-month followup showing erupted permanent mandibular left premolars.

After duration of 8 months, it was noted that underlying permanent mandibular premolars (34, 35) erupted into their normal position in the oral cavity, and a new bone formation was found in cystic lesion space (Figures 4 and 5).

## 5. Discussion

Radicular cysts originating from primary teeth are considered rare. The frequency is low because pulpal and periapical infections in deciduous teeth tend to drain more readily than those of permanent teeth and antigenic stimuli which evoke the changes leading to formation of radicular cyst may be different [1].

According to Mass et al. [5] the prevalence rate of radicular cysts associated with primary molars is probably higher compared with that in the reported literature. It is possible that, unlike cysts of permanent dentition, primary teeth are extracted but not submitted for pathological examination, a fact that may account for the low estimation of the real frequency of cysts associated with primary teeth [7].

As these cysts are asymptomatic till secondarily infected, they are usually diagnosed during routine radiographs. The sequelae of an untreated or undiagnosed radicular cyst could be harmful to the patient's future dental development. A patient with an untreated radicular cyst may present with the following consequences: swelling, tenderness, tooth mobility, and a bluish tinge caused by buccal expansion of the cortical plates. Furthermore, displacement of the successor tooth or, even more unforgiving, the loss of its vitality may result [4, 9, 10].

In the present case conservative treatment of the cyst that is marsupialization rather than enucleation is considered to save the premolar tooth bud and monitoring the eruption of the tooth.
